# Synthesis of Printable Polyvinyl Alcohol for Aerosol Jet and Inkjet Printing Technology

**DOI:** 10.3390/mi12020220

**Published:** 2021-02-22

**Authors:** Mahmuda Akter Monne, Chandan Qumar Howlader, Bhagyashree Mishra, Maggie Yihong Chen

**Affiliations:** 1 Materials Science, Engineering, and Commercialization Program, Texas State University, San Marcos, TX 78666, USA; mam638@txstate.edu (M.A.M.); chandan.howlader@txstate.edu (C.Q.H.); b_m415@txstate.edu (B.M.); 2 Ingram School of Engineering, Texas State University, San Marcos, TX 78666, USA

**Keywords:** aerosol jet printing, dielectric, etching, inkjet printing, polyvinyl alcohol (PVA), polymers, sacrificial material, micro-electro-mechanical-system (MEMS)

## Abstract

Polyvinyl Alcohol (PVA) is a promising polymer due to its high solubility with water, availability in low molecular weight, having short polymer chain, and cost-effectiveness in processing. Printed technology is gaining popularity to utilize processible solution materials at low/room temperature. This work demonstrates the synthesis of PVA solution for 2.5% *w*/*w*, 4.5% *w*/*w*, 6.5% *w*/*w*, 8.5% *w*/*w* and 10.5% *w*/*w* aqueous solution was formulated. Then the properties of the ink, such as viscosity, contact angle, surface tension, and printability by inkjet and aerosol jet printing, were investigated. The wettability of the ink was investigated on flexible (Kapton) and non-flexible (Silicon) substrates. Both were identified as suitable substrates for all concentrations of PVA. Additionally, we have shown aerosol jet printing (AJP) and inkjet printing (IJP) can produce multi-layer PVA structures. Finally, we have demonstrated the use of PVA as sacrificial material for micro-electro-mechanical-system (MEMS) device fabrication. The dielectric constant of printed PVA is 168 at 100 kHz, which shows an excellent candidate material for printed or traditional transistor fabrication.

## 1. Introduction

In 1915, Poly(vinyl alcohol) was discovered by F. Klatte from the precursor Poly(vinyl acetate) [1]. After that, the preparation of PVA was firstly described by W. O. Herrmann and W. Haehnel in 1924 [2,3,4]. However, it cannot be prepared with traditional polymerization because its monomer vinyl alcohol is not stable and rearranges readily to acetaldehyde. It is usually manufactured from hydrolysis of polyvinyl acetate, which involves the partial or total replacement of the ester groups of vinyl acetate by hydroxyl groups under defined conditions. Later, the PVA is precipitated, washed, and finally dried. The PVA properties depend on the polymer chain’s length or the degree of polymerization and the degree of hydrolysis [5,6]. PVA is an example of a water-soluble semi-crystalline synthetic polymer, and it is also slightly soluble in ethanol. It is important to mention that the higher the degree of hydrolysis and polymerization, the lower the solubility of PVA in cold water. Polyvinyl alcohol is a semi-crystalline, non-toxic, water-soluble, and biodegradable polymer. It is also biocompatible with human tissues and has excellent gas-barrier properties [7]. PVA has many applications, including but not limited to the manufacturing of cleaning and detergent products, in the food packaging industry, water treatment, textile, agriculture, and construction [7,8,9,10,11]. It also has recently attracted an increasing amount of attention for pharmaceutical uses (i.e., drug delivery) and in medical applications (e.g., wound dressing, soft contact lenses, and eye drops) [12,13,14,15]. PVA films can be produced in either a melt or solution form. Melt processing is compatible only with low hydrolysis or heavily plasticized PVA. At the same time, a PVA film can be deposited from the solution form through drop casting, spin coating, and electrospinning, etc. [16,17,18]. However, these methods have a few limitations in terms of waste of material. In case of spin coating, it has been reported that about 95% of material is wasted with no design/features/patterning capability [19,20,21]. Our work reports the synthesis of PVA and the potential of aerosol jet and inkjet printing technologies as a mean to provide a novel platform to produce multi-layer structures. This phenomenon will have a substantial impact on both the material processing and application perspectives.

Recent developments in the thin-film deposition sector have focused on cheap, simple, eco-friendly, and energy-saving processes. Printing is a modern fabrication process that fits perfectly within this framework. The significant advantages of printing technology are material utilization efficiency, mask-free and additive patterning, large area capability, compatibility with many substrates, and the low-cost fabrication process [22,23,24,25,26,27]. Printing technology is used to successfully deposit conductive and non-conductive nanomaterial, polymers, ceramic, 1D/2D materials, dielectric, biological, and pharmaceutical-based materials [28,29,30,31].

With the advancement of flexible electronics, low molecular weight and water-soluble elastic polymers as well as high permittivity dielectrics are in demand for MEMS device and flexible transistor fabrication [32,33,34,35,36,37]. Many polymers like polyvinylpyrrolidone (PVP), polymethylmethacrylate (PMMA), and PVA have been studied for their water solubility, electrical, and dielectric properties [38]. However, PVA is the most studied polymer due to its versatile properties. It is highly water-soluble, has a low cost, and has suitable film-forming properties. 

The selection of appropriate sacrificial material is a crucial part of the MEMS fabrication process. The sacrificial material must be deposited and finally removed without disturbing other layers. Along with developing printed technology, researchers have been working on selecting appropriate sacrificial materials for years. In [39], the use of sacrificial layer technique is discussed to create multilayer metalized structure using SU-8 dielectric. Multilayer suspended structure was successfully fabricated by adopting sputter coating, which is another form of solution processible technique. 

Additionally, the MEMS fabrication process is less standardized than other microelectronics fabrication processes. 3D printed MEMS is a potential candidate among researchers for a selection of less expensive materials and producing new structures for future applications [40]. However, it may be difficult to achieve sufficient resolution only with this technology compared to traditional MEMS fabrication technology [36,37,41]. A fundamental study has been conveyed to fabricate graphene or graphite-based flexible MEMS devices which enables future researchers to fabricate 2D material-based MEMS devices [42,43].

This study aims to present the synthesis of PVA solution for printing technology (aerosol jet and inkjet printing), characterize the solution, and present the application of PVA for MEMS and transistor device fabrication.

## 2. Experiments 

**Figure 1:**
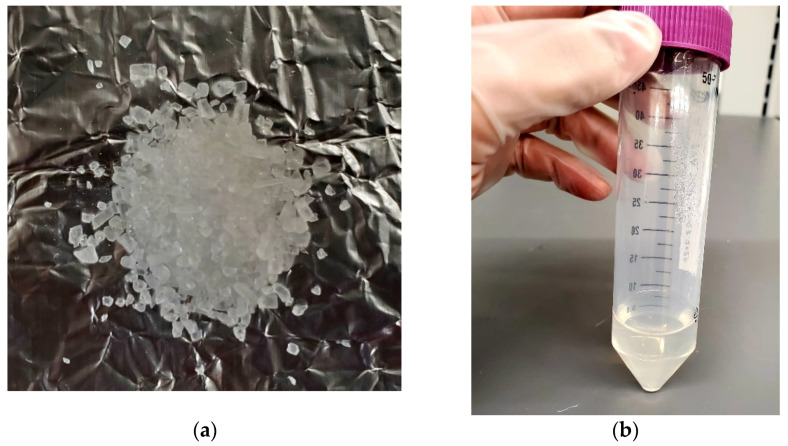
(**a**) The 98% hydrolyzed PVA crystal. (**b**) In-lab prepared PVA solution.

**Ink Formulation:** The PVA solution is synthesized by mixing low molecular weight (<400 kDa) PVA crystal with DI water and heating with a microwave. Different percentage of *w*/*w* PVA solutions were prepared for two printing techniques. Viscosity of the PVA solution was varied by changing the percent of PVA powder. It is observed that 0.5%–4.5% *w*/*w* PVA is good for inkjet printing technology and aerosol jet technology with the ultrasonic atomizer. The solution with >4.5% *w*/*w* PVA is suitable for aerosol jet printing with the pneumatic atomizer and other printing methods such as screen printing, spray coating, bar coating, blade coating, etc. To prepare a 2% *w*/*w* 10ml PVA solution, 10 mL of DI water is mixed with 0.2 g of low molecular weight PVA crystal and heated with a microwave. The microwave’s power setting is kept at 1000 watts, and the solution is microwaved for 180 s until a homogeneous solution is achieved. Later, the homogeneous solution is centrifuged for 30 min at 5000 rpm to eliminate the big and clustered polymer chains. Finally, the solution is filtered using a 0.25–0.45 µm filter before loading it to the printer reservoir. Figure 1 shows the 98% hydrolyzed PVA crystal from Sigma Aldrich (St. Louis, MO 63178, USA) and the in-lab prepared PVA solution. 

**Ink Characterization (Viscosity)**: Ink was prepared with 5 different concentrations; 2.5% *w*/*w*, 4.5% *w*/*w*, 6.5% *w*/*w*, 8.5% *w*/*w*, and 10.5% *w*/*w* PVA in DI water. An AMETEK Brookfield (DV2T) (Chandler, AZ 85225, USA) viscometer is used to measure the viscosity of each solution. Since the instrument gives multiple measured data for the viscosity measurement for different shear rates, so standard deviation of viscosity is added to Table 1. The standard deviation in Table 1 column 6 is among all the values of viscosity (at different set of shear rates) including minimum and maximum viscosity.

Viscosity was measured at 25 °C, with different spindle speeds and shear rate. Multiple shear rates were used due to various concentrations of PVA, which results in different viscosity. Usually, low-viscous inks need higher spindle speed and shear rate to get an accurate result. Table 1 shows a summary of viscosity measurement. 

**Contact Angle and Surface Tension (on Kapton and Silicon)**: Appropriate surface cleaning is a crucial step to acquire a good quality print. Substrates are cleaned in an air plasma for 15 mins to remove organic contaminants and physical ablation. The plasma cleaning process introduces chemical functional groups (carbonyl, carboxyl, hydroxyl) on the surface to make it hydrophilic. This paper also investigates the effect of plasma cleaning on contact angle. Since printable PVA is a water-based solution, a hydrophilic surface is essential to achieve a good quality print. The analysis of surface tension and contact angle is carried out to present the degree of hydrophilicity before and after the plasma cleaning process, which ultimately helped us decide hydrophilicity or hydrophobicity and the wettability of the printable PVA solution. The literature found that the surface tension in the range of (25–75) mN/m is adequate to achieve a good liquid tension toward the surface [40]. Through experimentation the surface tension is found to be between 30 mN/m to 50 mN/m for both the substrates. Since the expected surface tension is obtained without plasma treatment, thus plasma treatment is not carried out to measure the surface tension.

On the other hand, contact angle presents the wettability or surface coverage of a solution. The contact angle between 0°–90° is preferable to achieve a good quality print. However, a contact angle toward 0° will have high wettability, which means it will spread out easily after printing process. This solution is good to cover large area printing. In addition, the contact angle toward 90° will have less spreading issue and higher possibility of holding any structure.

**Figure 2:**
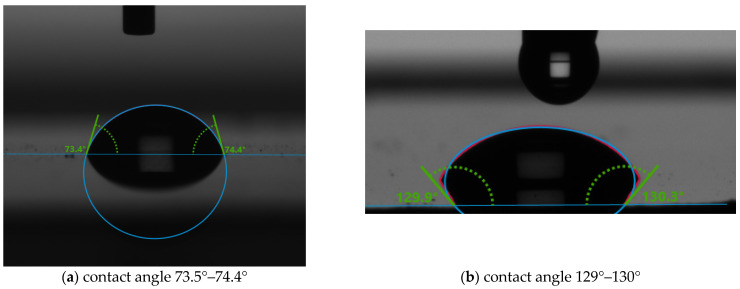
The contact angle of 2.5% PVA on Kapton (**a**) without plasma treatment and (**b**) with plasma treatment.

Surface tension and contact angle has been measured using a KRUSS Drop Shape Analyzer (DSA 1000) (Gulf Coast Region, TX 78373, USA). The contact angle and surface tension measurements were done at 20 °C and with a 2 mm syringe tip size. Two different substrates, Kapton and Silicon, were used to test for all five concentrations of PVA. In printed electronics, it is essential to check surface tension and contact angle for two reasons; (i) determine the bonding between solution and substrate material and (ii) determine if the solution can hold the structure. Figure 2 shows the contact angle of 2.5% *w*/*w* PVA on Kapton without and with plasma treatment. The contact angle is found to be 73.5°–74.4° for without O_2_ plasma-treated surface and 129°–130° for the plasma-treated one. It is to be noted that the substrate wettability is useful when the contact angle is between 0°- 90°. So, the Kapton substrates do not need plasma treatment to achieve good quality printing with PVA. 

**Figure 3:**
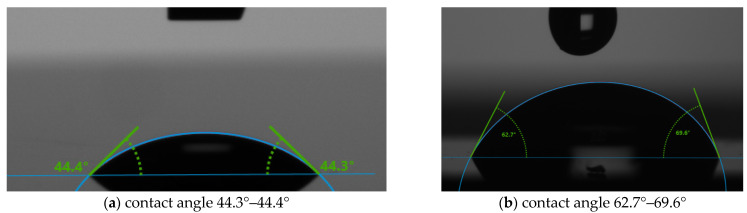
The contact angle of 2.5% PVA on Silicon (**a**) without plasma treatment and (**b**) with plasma treatment.

Figure 3 shows the contact angle of 2.5% PVA on Silicon, and the angle is found to be 44.3°–44.4° without O_2_ plasma-treated surface and 62.7°–69.6° for the plasma-treated one. From these two analyses, it is observed that the O_2_ plasma improves the contact angle of the substrate significantly.

**Figure 4:**
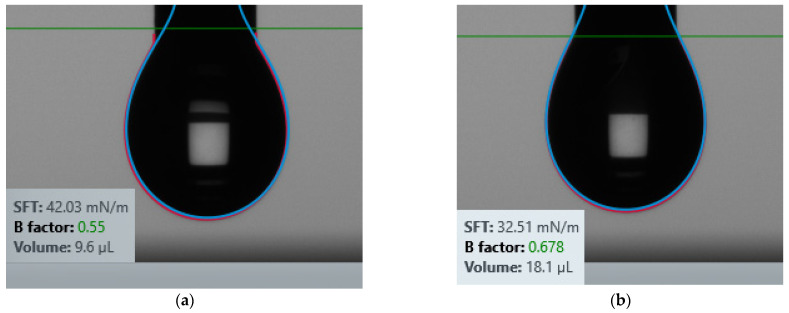
The surface tension of 2.5% PVA on (**a**) Kapton and (**b**) Silicon.

Figure 4 shows the surface tension of 2.5% PVA on Kapton and Silicon. The surface tension is found to be 42.03 mN/m for Kapton and 32.51 mN/m for Silicon substrate. To achieve a good inkjet printing with any material, the surface tension has to be in between (25–75) mN/m and viscosity has to be in between (1–8)cP [44]. 

Table 2 shows the summary of contact angles on Kapton and Silicon for all five concentrations of PVA. It is observed that both substrates show higher surface tension for higher concentrated or higher viscous PVA. In addition, the contact angle does not vary much for the Silicon substrate for all concentrated PVA while Kapton has the contact angle between 0°–90° without a plasma-treated surface, and above 100° for a plasma-treated one. It is possible to achieve good printing with both the substrates, only Kapton does not need plasma treatment. 

**Aerosol Jet Printing of PVA:** An aerosol jet AJ 300 system from the company Optomec was used to print a 5 mm by 5 mm pattern. The reason for printing a large pattern is to show the print-quality for large scale printing area. In this work, two different printing methods have been adopted to print a large area with AJP and small area with IJP. Furthermore, by developing an appropriate ink waveform and printing parameters, it is also possible to achieve both large and small area printing with both the deposition system. The aerosol jet system has two different atomizers called ultrasonic atomizer and pneumatic atomizer. The ultrasonic atomizer is used for the solutions that has a viscosity range of (1–5) cP, and the pneumatic atomizer is used for all of the kinds of solution that have a viscosity from (1–1000) cP. This article attempts to develop appropriate printing parameters and recipe to print with ultrasonic atomizer. 

During the ultrasonic atomization, the solution is agitated by a pressure wave of appropriate frequency and amplitude. When a suitable pressure wave is applied, a capillary wave structure forms at the liquid interface with a desire aerosol droplet dimension of 3 µm to 5 µm. This capillary wave looks like a standing wave, where crests of these waves break off to form the aerosol droplets. Different sizes of nozzles (e.g., 100 µm, 150 µm, 200 µm, 250 µm, and 300 µm) can be used with AJ 300 system. The polymer chains are tends to amalgamate, which is why, the 200 µm nozzle is used to deposit the film. The other printing parameters used during the deposition process are: printing speed 1 mm/sec, sheath gas flow 5 sccm, atomizer gas flow 50 sccm, atomizer current 627 mA, platen temperature 45 °C, and curing temperature 80–90 °C for 15 min. Table 3 shows the summary of the printing parameters for PVA deposition with ultrasonic atomizer.

**Figure 5:**
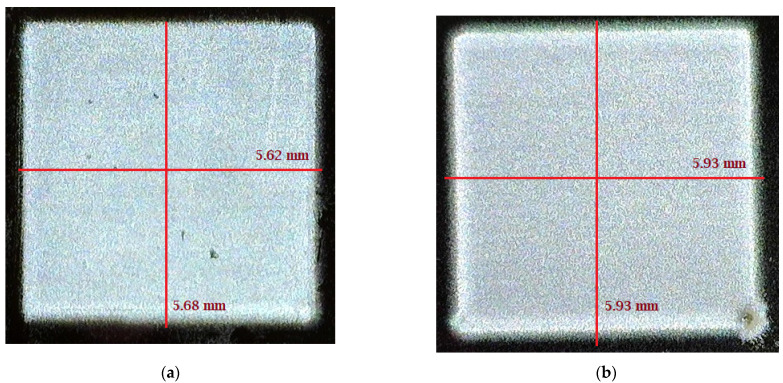
2.5% PVA printed on Silicon substrate: (**a**) Two printed layers; (**b**) four printed layers.

A test pattern size of 5 mm by 5 mm was designed using AutoCAD 2019 (Autodesk, San Rafael, CA 94903, USA). The file was saved as .dxf format. Then, using manufacturer provided plug-in, VMTools, the .dxf file was converted to .prg file. The .prg file is the only file format that is supported by the AJ 300 deposition system. A multi-layer structure is printed on a Silicon substrate. Figure 5 shows the two layers and four layers of PVA printed patterns on the substrate. Although, the target length and width are 5 mm by 5 mm, however, after printing the pattern size is 5.62 mm by 5.68 mm, and 5.93 mm by 5.93 mm for two and four PVA layers, respectively. It is to be noted that, by appropriate pattern modification, it is possible to achieve the target pattern size. The maximum size of pattern that can be printed by an AJ 300 is 300 mm by 300 mm with the highest in-situ platen temperature of 120 °C. For large sized or multi-layer pattern printing, the ink spreading will increase along with time. 

**Figure 6:**
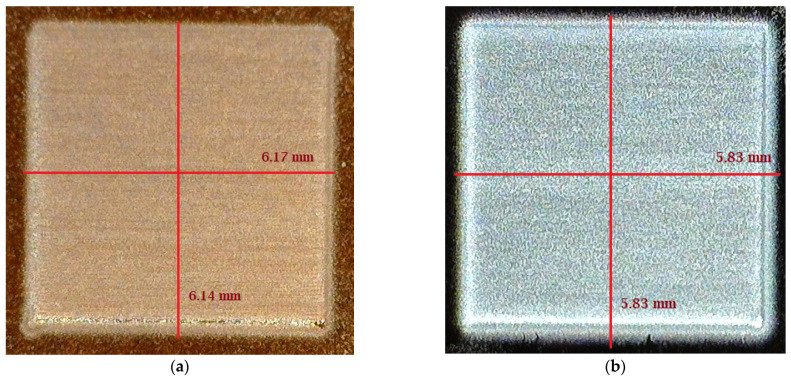
2.5% PVA printed on Kapton and Silicon. (**a**) 5 mm × 5 mm targeted pattern printed on Kapton; (**b**) 5 mm × 5 mm targeted pattern printed on Silicon.

2.5% PVA was also printed on a Kapton substrate. Figure 6 shows the 5 mm by 5mm pattern printed on both Kapton and Silicon substrate. Both have three printed layers, but after printing, 6.17 mm by 6.14 mm was found for Kapton, and 5.83 mm by 5.83 mm was found for the Silicon substrate. So, it can be concluded that the Silicon substrate is superior to the Kapton substrate from the print-quality perspective.

**Figure 7:**
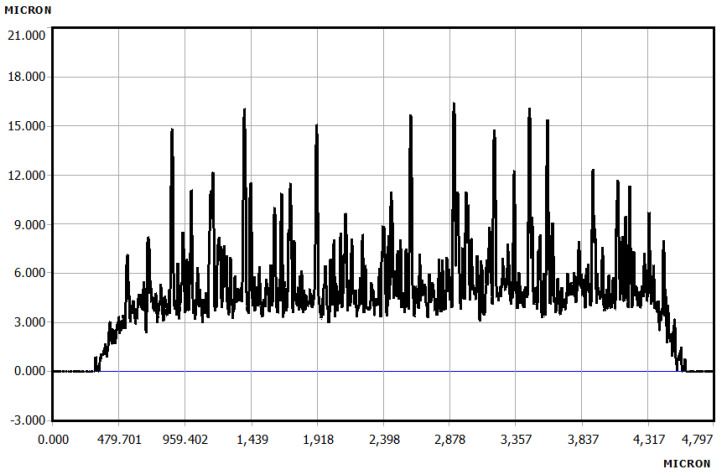
The thickness and roughness profile of 2.5%, and four printed layers of PVA.

**Thickness Profile of Printed PVA:** KLA Tencor D-300 (Milpitas, CA 95035, USA) profiler was used to measure the thickness and roughness profile of the printed patterns. Figure 7 shows the thickness and roughness profile of four printed layers of 2.5% PVA on the Silicon substrate.

The average thickness and roughness were found to be 5.7 µm and 1.8 µm, respectively. Table 4 shows the summary of thickness and roughness profiles for different printed layers on Silicon. It is observed from the table that the spreading and roughness both increased with the increment of printed layers. 

**Inkjet Printing of PVA:** The Fujifilm Dimatix DMP-2800 (Santa Clara, CA 95050, USA) deposition system allows fluidic materials on 8 by 11-inch substrate with a maximum substrate height of 25 mm. The deposition head utilizes a disposable piezo inkjet cartridge. The platen of the system is vacuum controlled with an adjustable temperature up to 60 °C. The ink-dispensing mechanism by piezo inkjet cartridge head is activated by four different voltage phases, which can be adjusted up to 40 V to achieve good jetting. The voltage phases break down a solution into droplets to start printing/jetting process. It is very difficult to handle long chain polymers with inkjet printing because the highest voltage of DMP-2800 cannot break the long polymer chains to make it into droplets form. However, PVA is a short chain polymer, hence it is possible to break it’s polymer chain with the voltage. Although, due to the amalgamation tendency, the nozzles tend to get clogged very frequently. PVA test print is carried out for two different types of pattern: For smaller area coverage and for larger area coverage, (i) 5000 µm × 150 µm line and (ii) 5000 µm × 5000 µm square, respectively.

**Figure 8:**
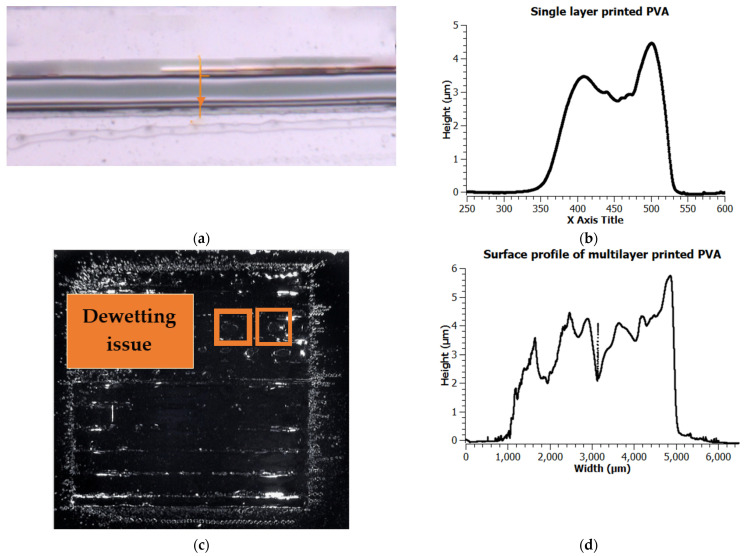
Printed PVA on Silicon substrate (**a**) (ii) 5000 µm × 150 µm line. (**b**) Thickness of the line; (**c**) 5000 µm × 5000 µm square. (**d**) Thickness of the square.

Figure 8 shows the inkjet printed PVA on a Silicon substrate. Figure 8a is the single-layer printed line with an average thickness of 3.5 µm and roughness (Ra) of 0.84 µm (Figure 8b). Additionally, Figure 8c shows the multilayer printed PVA with an average thickness of 5.5 µm and roughness (Ra) of 1.14 µm (Figure 8d). It is observed that the printed PVA has perfect coverage for the smaller area with low surface roughness, and it has some dewetting and increased roughness issues for larger area coverage. 

**Figure 9:**
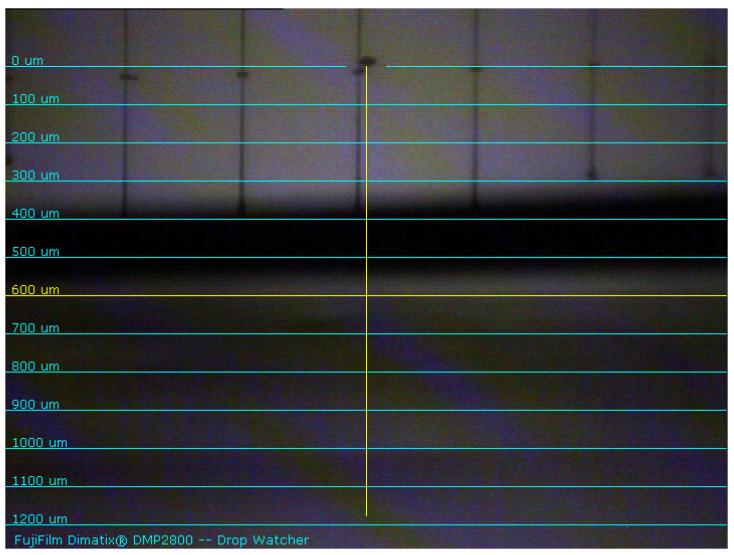
Jetting condition of PVA from the nozzles 5 to 10.

The recipe used to start jetting the PVA is the default Fujifilm Dimatix jetting waveform. Figure 9 shows the jetting condition of PVA solution with the drop watcher of DMP-2800. The nozzles showed here are from 5 to 10 with a jetting velocity of about 4 m/sec. In Table 5, it is seen that the thickness of the single layer deposited PVA is about 3.5 µm. We have used only a single printed layer of 2.5% PVA solution to create a suspended structure for MEMS device application. Typically for a standard MEMS device, the distance between the top and bottom electrode should be (0.5–4) µm. Ideally, the lower the gap between top and bottom electrode, the lower the actuation voltage. In this work, with 2.5% PVA solution one printed layer of PVA gives about 3.5 µm thickness, and that is why the print is limited to single layer only. For the concentrations > 2.5%, the thickness is assumed to be > 4 µm. Thus, higher concentration of PVA is not taken into consideration to create the suspended structure for the proposed application.

The other parameters used to achieve good jetting is presented in Table 6. The parameter values for nozzle size, jetting frequency, nozzle voltage, platen temperature, cartridge temperature, and meniscus is 10 pL, 2 kHz, 40 V, 50 °C, 30 °C, and 3, respectively.

**Figure 10:**
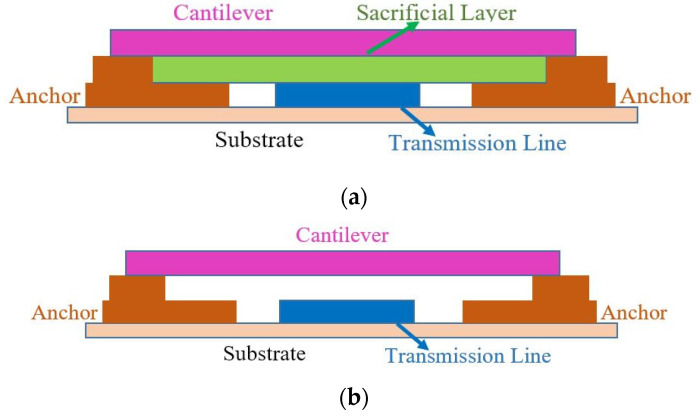
The schematic of a typical double clamped micro-electro-mechanical-system (MEMS) device structure. (**a**) MEMS device structure before the removal of sacrificial layer; (**b**) MEMS device structure after the removal of sacrificial layer.

**PVA as Sacrificial Material for MEMS Device Fabrication:**Figure 10 shows the schematic of a typical double clamped MEMS device where (a) shows the device schematic with sacrificial layer and (b) is the final device after removing the sacrificial layer. The sacrificial layer is an essential part of MEMS device fabrication and it is not a part of the final device. This layer is used to make a suspended MEMS cantilever structure, then removed by an etching process. In this work, printed PVA is introduced as a good alternative to other sacrificial materials to make a suspended structure for the MEMS cantilever application.

It is to be noted that, this work does not present a complete MEMS device structure. It only shows how PVA can be used as a material that can help create a suspended structure using a safe and simple etching process. 

**Figure 11:**
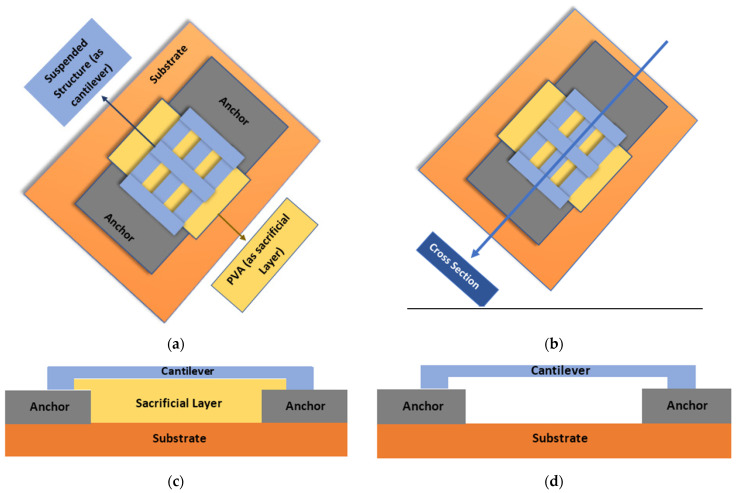
Schematic of a suspended structure where printed PVA used as a sacrificial material. (**a**) schematic and top view of the suspended structure; (**b**) the region from where the cross-section was taken, (**c**) structure before the final removal of the PVA sacrificial layer and (**d**)final suspended structure after the removal of sacrificial layer.

Figure 11a shows the top view and the schematic that is used to make a suspended structure. The cross-section region is illustrated in Figure 11b, while Figure 11c,d show the elaborated cross-section view of the structure before and after the removal of sacrificial layer. It is important to highlight that this structure is not a complete MEMS structure; here the printed PVA is portrayed as a sacrificial material which can be used in MEMS device fabrication in future work. 

## 3. Results

**Etching of PVA Layer:** A DMP-2800 deposition system can handle materials with viscosity up to 12 cP, which is why 2.5% PVA solution is chosen to deposit sacrificial layer. A single layer of PVA with a thickness of 3.5 µm is deposited in between a top mesh suspended structure (as cantilever) and bottom anchors as clamper. Later, the PVA layer is removed using only hot DI water (80 °C–90 °C) without disrupting Ag layers. Table 7 shows the etching time and rate of 2.5% PVA layers with different thicknesses. It is to be noted that, with the increase of thickness, etching time increased and etching rate decreased due to the creation of strong polymeric bonding.

**Figure 12:**
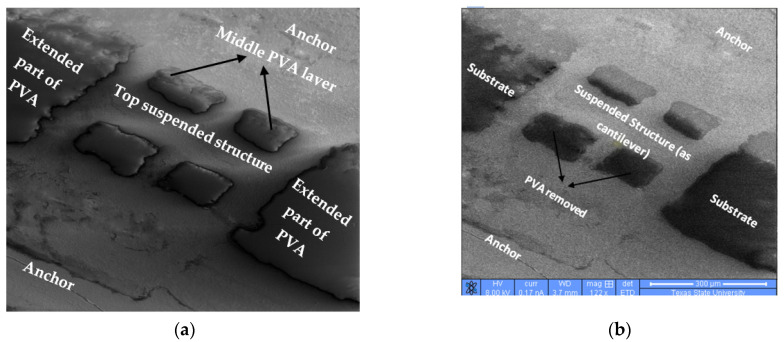
Mesh Cantilever structure (**a**) before PVA removal and (**b**) after PVA removal.

PVA is used as a sacrificial material to create a free-standing mesh structure. Figure 12 shows the printed mesh structure (a) before and (b) after the removal of middle PVA layer. The FEI Helios NanoLab 400 Dual Beam system (Hillsboro, OR 97124-5793, USA) with a fully digital Field Emission Scanning Electron Microscope (FE SEM) is used to take the imaging. An Everhart–Thornley detector with a voltage, current, and magnification of 8.00 kV, 0.17 nA, and 122 x, respectively, is used during imaging. A higher current and exciting voltage would improve the imaging quality; however, a low exciting current is used since PVA is a non-conductive polymer and higher current will destroy the sample.

The SEM image shows a mesh double clamped suspended cantilever structure where printed PVA is sandwiched between top mesh structure and bottom double clamped anchors. In Figure 12a, the printed PVA is between mesh structure and anchors. In this image, PVA is deposited between top mesh structure (where mesh structure deposited by solution processible silver ink) and bottom anchor structure (anchors also deposited by silver ink). The substrate is not visible at all, because extended part of deposited PVA is covering the substrate region. It is important to emphasize that the four windows are not precise enough, not because of the non-accurate deposition of PVA layer, but because the windows are designed too small (50 µm by 50 µm) and it is very challenging to fabricate these small features with printing technique. The spreading of printed silver is responsible for the non-uniform squares. On the other hand, Figure 12b shows the suspended and free-standing mesh structure after the fully removal of PVA sacrificial layer. Additionally, in this image, substrate is also fully visible as the PVA layer is completely gone due to water etching.

It is to be noted that the mechanical structure or the cantilever of MEMS devices must be free-standing as this will be a movable part. PMMA is the most widely used sacrificial material for traditional MEMS fabrication techniques. However, PMMA is a long-chain polymer, and it is not suitable for printing technology. PVA is a short-chain polymer with low molecular weight; thus, PVA is used and suggested as a good alternative of PMMA as sacrificial material for printing technology. 

**PVA as Dielectric Material for Transistor Fabrication:** A Hermes Mercury Probe 802–150 (Materials Development Corporation, Chatsworth, CA 91311, USA) is used to measure the dataset. The equipment measures capacitance of thin film on Si wafer using a mercury dot to form another electrode. It creates a MOS capacitor. From there, the dielectric constant is calculated using the following formula,

(1) $$C=\epsilon_{0}\epsilon_{r}Area/Thickness$$

where $\;\epsilon_{r\;}$ is the dielectric constant.

The printed thin film of PVA on Si wafer acts as metal-oxide-semiconductor (MOS) capacitor when attached to Hermes equipment’s probe and back contact. When the DC voltage sweep is applied during CV measurement at low frequency, the MOS capacitor has depletion, accumulation, and an inversion zone. 

**Figure 13:**
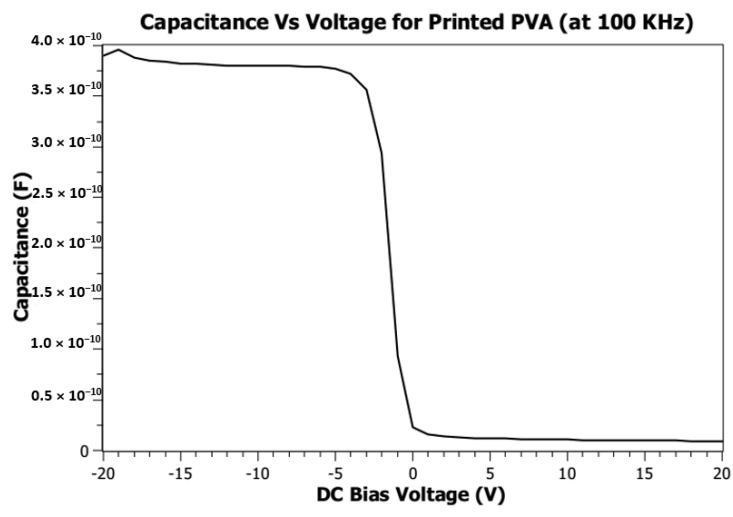
Capacitance Vs. bias voltage measurement.

The dielectric constant of printed PVA has been calculated from capacitance vs. DC bias voltage measurement. Figure 13 shows the capacitance vs. DC bias voltage at 100 kHz. The DC bias voltage is applied from −20 V to 20 V, and the highest capacitance measured is 4 × 10^−10^ F. From that measurement, the dielectric constant of printed PVA is calculated to be 168.

## 4. Conclusions

We have demonstrated the synthesis, characterization, and application of printable PVA by Aerosol Jet Printing (AJP) and Inkjet Printing (IJP) technologies. Five different percentage of PVA of 2.5% *w*/*w*, 4.5% *w*/*w*, 6.5% *w*/*w*, 8.5% *w*/*w*, and 10.5% *w*/*w* aqueous solution were formulated and characterized with respect to viscosity, contact angle, surface tension, and printability. We have demonstrated that the PVA solution can be used to produce a multi-layer structure with patterning by AJP and IJP printing technology. This polymer is also promising to use as a sacrificial material for MEMS device fabrication. Additionally, the dielectric constant of printed PVA film is 168 at 100 kHz, which means it can be an excellent candidate material for printed or traditional transistor fabrication.

## Figures and Tables

**Table 1:** Viscosity measurement of all five PVA solution.

PVA Percentage (%)	Spindle Speed (rpm)	Shear Rate (1/s)	Viscosity Minimum (cP)	Viscosity Maximum (cP)	Standard Deviation for Viscosity (cP)
2.5	50–200	375–1500	4.93	5.00	0.03
4.5	25–100	187.5–750	8.80	9.00	0.08
6.5	15–50	112.5–375	24.2	25.5	0.5
8.5	10–25	75–187.5	34.8	35.3	0.3
10.5	10–25	75–187.5	81	82	1

**Table 2:** The summary of the contact angle on Kapton and Silicon without surface treatment.

PVA Concentration (%)	Contact Angle (Kapton)	Contact Angle (Silicon)	Surface Tension (Kapton) (mN/m)	Standard Deviation for Surface Tension (Kapton) (mN/m)	Surface Tension (Silicon) (mN/m)	Standard Deviation for Surface Tension (Silicon) (mN/m)
2.5	73.5°–74.4°	44.3°–44.4°	42.0	0.6	32.5	0.6
4.5	74.5°–75°	44.3°–45.2°	45.5	0.3	39	2
6.5	77.6°–78.5°	45.3°–45.5°	46	1	44.9	0.9
8.5	80°–82.3°	45.4°–45.9°	48.8	0.8	47	2
10.5	85.2°–85.5°	46.3°–46.6°	50.4	0.8	49.6	0.9

**Table 3:** The summary of the printing parameters for PVA deposition with ultrasonic atomizer.

Atomizer	Nozzle Size (µm)	Printing Speed (mm/sec)	Sheath Gas Flow (SCCM)	Atomizer Gas Flow (SCCM)	Atomizer Current (mA)	Platen Temperature (°C)	Curing Temperature (°C)
Ultrasonic	200	1	5	50	627	45	80–90

**Table 4:** Thickness and roughness profiles for different printed layers of PVA.

Printed Layers (on Silicon)	Targeted Pattern (mm × mm)	Achieved Pattern (mm × mm)	Thickness (µm)	Roughness (µm)
1	5 × 5	5.44 × 5.44	1.8	0.45
2	5 × 5	5.62 × 5.68	2.53	0.7
3	5 × 5	5.83 × 5.83	3.73	0.86
4	5 × 5	5.93 × 5.93	5.68	1.8

**Table 5:** Summary table of characterized printed PVA.

Targeted Pattern	Achieved	Printed Layers	Thickness (µm)	Standard Deviation for Thickness (µm)
5000 µm × 150 µm line	5250 µm × 190 µm line	1	3.5	0.8
5000 µm × 5000 µm square	5255 µm × 4060 µm square	2	5.5	1

**Table 6:** Additional inkjet printing parameters to print PVA.

Nozzle Size	Jetting Frequency	Nozzle Voltage	Platen Temperature	Cartridge Temperature	Meniscus
10 pL	2 kHz	40 V	50 °C	32 °C	3

**Table 7:** Etching rate for PVA layers of different thicknesses.

PVA Layer Thickness (µm)	Etching Solution	Etching Time	Etching Rate
1	DI water with 110 °C	40 s	0.025 µm/s
4.5	DI water with 110 °C	~7 min	0.714 µm/min
9.5	DI water with 110 °C	~15 min	0.66 µm/min
15	DI water with 110 °C	~25 min	0.6 µm/min
23	DI water with 110 °C	~37 min	0.55 µm/min
